# S-map parameters for APSIM

**DOI:** 10.1016/j.mex.2022.101632

**Published:** 2022-02-06

**Authors:** Iris Vogeler, Linda Lilburne, Trevor Webb, Rogerio Cichota, Joanna Sharp, Sam Carrick, Hamish Brown, Val Snow

**Affiliations:** a The New Zealand Institute for Plant & Food Research Limited, Lincoln, New Zealand; b Manaaki Whenua – Landcare Research, New Zealand; c AgResearch – Lincoln Research Centre, New Zealand; d Aarhus University, Tjele, Denmark

**Keywords:** Process-based modelling, Digital spatial soil information, Sensitivity analysis

## Abstract

Agroecosystem models have become an important tool for impact assessment studies, and their results are often used for management and policy decisions. Soil information is a key input for these models, yet site-specific soil property data are often not available, and soil databases are increasingly being used to provide input parameters. For New Zealand, the digital spatial soil information system S-map provides geospatial data on a range of soil characteristics, including estimates of soil water properties. We describe a protocol for how properties from S-map can be used as input parameters for the APSIM (Agricultural Production Systems sIMulator) framework. Finally, we investigate how changes in the physical description of soil layers, and soil organic matter pools, affect the various outputs of APSIM.

• This paper presents a description of how information from S-map, a digital soil map of New Zealand, can be used for building a soil description for APSIM.
• A sensitivity analysis shows the effect of soil layering and the set-up setup, size, and distribution of SOM pools on model outputs, including plant growth and N leaching

## Highlights

• Listing and describing the parameters needed to build a soil description in APSIM based on S-map
• Sensitivity analysis of physical and chemical soil parameterisation
• For regional or national assessments the use of S-map information is appropriate

## Graphical abstract

**Figure:**
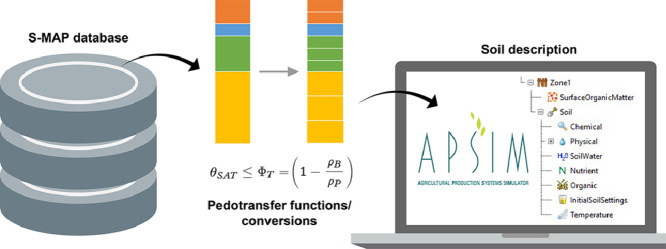

Specification table

**Table:** 

Subject Area:	Environmental Science
More specific subject area:	Agroecosystem modelling
Method name:	S-map parameters for APSIM
Reference for original method:	Dalgliesh, N.; Hochman, Z.; Huth, N.; & Holzworth, D. 2016. A protocol for the development of APSoil parameter values for use in APSIM – Version 4, CSIRO, Australia. 24 p.Cichota, R., Vogeler, I., Sharp, J., Verburg, K., Huth, N., Holzworth, D, Dalgliesh, N., Snow, V. A protocol to build soil descriptions for APSIM simulations. MethodX, submitted.
Resource availability:	www.apsim.info/documentation/model-documentation/soil-modules-documentationS-Map Online | Manaaki Whenua - Landcare Research

## Introduction

Growing concerns about the negative environmental effects of crop production systems, climate change, and food security have increased the use of process-based crop models to help decision-making at local, regional, and global scales. Apart from on-farm decisions regarding nutrient cycling and nutrient use efficiency, crop models are being used to evaluate the potential of crops, cultivars, and their management under different and changing agro-climatic environments, as well as for policy development.

Soil data are key input parameters for biophysical crop models and are important for accurate simulations of yield [1]. Depending on the complexity and the processes, which are simulated within a particular model, soil data requirements vary. Information is required based on either layers or soil horizons. This information should be site-specific and can be obtained by soil sampling or through the use of soil databases. Site-specific data are superior for guiding on-farm decision-making, whereas the use of more general information from soil survey databases is often used for larger areas (e.g., region and sub-catchment to national-scale modelling). For example, soil databases are often used for modelling the sensitivity of crops to changing climates [2] and for land-use suitability. Some soil databases have been compiled at the global scale, including the World Soil Information Service (WoSIS; [3]), the GlobalSoilMap [4], and the Harmonized World Soil Database [5]. Such global-scale data are rather coarse, and, as shown by [6], cannot be used to accurately simulate crop yields. In particular, simulated crop yields were shown to be sensitive to soil hydraulic properties. Another option for regional modelling is the use of a hydrological classification system link soil properties to soil hydrology, as recently done by [7], who used the FOOTPRINT soil type classification system, plus information of topsoil soil texture and organic matter contents from a digital soil map for regional scale modelling of herbicide leaching with the MACRO-SE model.

Databases at the national scale often capture the spatial variability of soil properties in a country better than global databases. For New Zealand (NZ), S-map, a digital spatial soil information system, has been developed [8]. One component of S-map is the dynamic (i.e., on-demand) generation of soil factsheets from the S-map database. These factsheets contain, among other things, estimates of the soil water properties of a specified soil. This information is valuable for setting up hydrological and cropping models, such as the Agricultural Production Systems sIMulator (APSIM; [9,10]). A current constraint when using S-map to set up APSIM simulations is a standardised methodology for how information from S-map should be entered in the soil model of APSIM. Such a standardised methodology assures confidence in the model output, and in any guidelines or policies that might be developed from this.

The main objective of this paper is to define a basic protocol for setting up soil descriptions for APSIM based on S-map data and some additional information from other national sources. Also, a sensitivity analysis of various model outputs to the set-up of the soil description is provided to assist and guide future users. This includes soil layering, soil hydraulic parameters, and initial values for the soil organic matter pools.

## Overview of APSIM

APSIM is a modelling framework designed and maintained by the APSIM Initiative. A detailed description of the various models within the APSIM framework can be found on the website (www.apsim.info). An abridged description of the models used to simulate soil processes in APSIM is provided in [11]. The paper also lists and describes the basic soil inputs (including physical and chemical characteristics), and how to enter specific parameters in the interface, based on measurements or pedotransfer functions. Currently there are two versions of APSIM available: APSIM *Classic* is several decades old, with dated infrastructure, and is gradually being recoded as APSIM *Next Generation*, which retains much of the earlier functionality but with a modern and more maintainable code base. Both versions contain the same soil models and mostly use the same soil parameters, but the user interface varies somewhat. For modelling soil water movement and solute transport, two different approaches are available for most modelling purposes: SoilWater (also called SoilWat; [12]) and SWIM3 [13]). (SWIM2 is only recommended for very experienced APSIM users and its parameterisation is not described here.) SoilWater is based on the tipping bucket approach, and SWIM3 on the Richards’ equation and the convection dispersion equation. These models differ in the approach and level of complexity used to describe the relevant processes. They have many parameters in common but others that differ, as described below.

Within the APSIM interface, a simulation is a collection of nodes, with the soil node being one of the fundamental components of an APSIM simulation. The soil node is itself a container, with several further child nodes, which comprise entry points for general soil parameterisation (layer structure, physical and chemical properties, etc.), as well as for setting up model-specific parameters (SoilWater, SoilTemperature). Although most parameters are the same in the *Classic* version and in APSIM *Next Generation*, due to some differences between the two versions the methodology for soil is provided here in broad groups (physical properties, chemical properties, soil organic matter, etc.).

## The New Zealand soil classification system and S-map

A digital spatial soil information system (S-map; smap.landcareresearch.co.nz; [14]) has been developed to provide information on NZ soils based on data from various sources (maps, survey measurements, etc.). Among other uses, S-map provides soil information required by simulation models, such as OVERSEER® and APSIM [15,16]. The S-map classification is based on the New Zealand Soil Classification (NZSC), developed in the 1980s, which organises the soils of NZ according to a hierarchy of five different levels: soil order, group, subgroup, family, and sibling [17,18]. The classification into soil orders is based on various factors in their formation: age, climate, soil hydrology, and parent material [19]. There are 15 different soil orders: Raw Soils (W), Recent Soils (R), Anthropic Soils (A), Semiarid Soils (S), Pallic Soils (P), Brown Soils (B), Podzols (Z), Gley Soils (G), Organic Soils (O), Melanic Soils (E), Pumice Soils (M), Allophanic Soils (L), Ultic Soils (U), Granular Soils (N), and Oxidic Soils (X). Each order is further sub-divided into group and sub-group based on diagnostic horizons and other features.

At the fourth level, the grouping into soil families is based on the soil profile material and form, rock type, texture, and permeability. Soil families are given a geographical name, but are also identified with a four- or five-character abbreviated name. At the fifth level, the soil families are further refined into soil siblings, each corresponding to unique combinations of drainage class, topsoil stoniness, soil depth, texture contrasts, and functional horizons. Each S-map sibling is described by a stack of up to six functional horizons. The concept of functional horizons has been developed by Webb [20], and was based on the relationship between various morphologic characteristics (ped size, ped surface features, and soil packing class) and saturated hydraulic conductivity (*K*s) in a range of NZ soils [21]. This concept of functional horizons was extended to a NZ-wide data set encompassing a wide range of soils [22] and to inform the prediction of a number of soil physical properties.

Soil mapping in S-map is at a nominal scale of 1:50,000. This resolution means there are commonly two or more soil siblings associated with a selected location (map polygon). When using S-map information for model parameterisation, it is therefore recommended to consider the characteristics of each of the identified siblings in a selected location, and their proportion, and select the S-map sibling that best matches field observations. If no local information is available, the dominant S-map sibling (i.e., the first-listed sibling) should be used. Alternatively, it may be useful to simulate each of the siblings identified in a map polygon. S-map contains information to a depth of 1 m. If deeper soil profiles are to be simulated (which is recommended, as plant roots often have a deeper rooting depth), the bottom functional horizon should be extended to 2 m, unless separate information on deeper layers is available, such as the presence of an impermeable layer or an increase in the proportion of gravels, or expert knowledge based on the position in the landscape. While this extension could result in incorrect representation of the soil profile with large uncertainties, measured data on deeper soil layers are rarely available.

## Defining soil parameters using S-map

This section describes a protocol for using soil information from S-map for setting up soils in APSIM. Excluded from the description are organic soils, because these have not been tested in APSIM for the correctness of the various parameter values and their effect on model outputs. Not all of the required soil information is provided by S-map, so users are referred to Cichota *et al.* (submitted) for guidance on the additional parameters. When using S-map, the various soil parameters can manually be entered into APSIM using factsheets downloaded from S-map Online. While it is possible to set up a new soil from scratch, it is easier to modify an existing one from another simulation, or from the APSoil database, a repository of soils information developed for use in the APSIM framework [23]. The modified soil can then be saved under a new name. Note that soils modified in this way do not enter the public domain as part of the official APSoil database (a protocol to add a soil parameterisation to APSoil can be found at www.apsim.info/apsim-model/apsoil). Alternatively, a ready-built APSIM soil library can be generated on demand using a custom web processing service (WPS) by arrangement with Manaaki Whenua – Landcare Research. The latter avoids the time-consuming and error-prone manual parametrisation process. Generating a library using the WPS involves entering a list of soil sibling names, the desired soil water model (SoilWater or SWIM3), the crops of interest, and the previous land use.

### Base soil node interface

In the soil node some general descriptors for the soil should be entered, such as the location (geographical coordinates), the soil type or name, the soil texture, and, if based on S-map, the sibling name.

### Layer structure and depth

S-map specifies soil parameters to a depth of 2 m, based on data that generally only goes to 1 m. For both SoilWater and SWIM3, the layers are aligned with the functional horizon boundaries listed in S-map, but the layering is different for the two models, as dictated by their solution methods. For SoilWater, the maximum layer thickness is 200 mm for the top layer and 600 mm for the remaining soil profile. If a functional horizon exceeds those values, the horizon is evenly split into two layers using the LayerStructure node, and if it exceeds 900 mm, it is split into three. For SWIM3, the layers have smaller depth increments, particularly at the top and bottom of the profile. For the top functional horizon, the soil is split into layers of defined thickness (mm) following the sequence: 10, 20, 30, 40 mm, with the remainder split into 100 mm increments. For example, if a top horizon is 80 mm, it will be split into four layers of thickness 10, 20, 30, and 20 mm. Below the top functional horizon, a layer is added for each functional horizon, but if any of the horizons is more than 150 mm, it is split into equal-sized, smaller layers of between 75 and 150 mm. The last functional horizon is handled differently, because it is extended down to the bottom of the soil profile and so will usually be greater than 1 m thick. Two layers of 50 mm and then a 20 mm layer are split off at the bottom of the last functional horizon. In SWIM3, a zero matric potential gradient is generally assumed to exist below the bottom boundary. Alternativly, a (fluctuating) water table can be added, as well as subsurface drains.

### Soil chemical properties

Various chemical parameters that are routinely measured and used (e.g., pH, CEC, nutrient content, etc.) should be specified within the Analysis and Initial nitrogen nodes in APSIM *Classic*, or the Chemical node in APSIM *Next Generation*. Although some of these values are currently not used by any of the common models, they could be important in the case of further model refinement and development.

#### Soil pH

S-map does not currently provide pH values for the siblings. A neutral value, between 6.0 and 7.0, can be used to make pH non-limiting for processes such as mineralisation and denitrification. Alternatively, values can be obtained from measurements or a pH prediction model such as that used by Roudier, Burge [24], which contains pH estimates for all of New Zealand on a 100 m × 100 m grid. The values were derived from a range of spatial predictors, including land use, topography, and climate layers, but do not include the effects of local management such as liming. Note that APSIM does not vary the initial pH during the simulation.

#### CEC

The CEC in S-map is a weighted sum of the percentage organic carbon, percentage clay, and percentage silt found in each functional horizon following Curtin and Rostad [25]:

$$CEC=2.25f_{OC}+0.267f_{C}+0.067f_{S}$$

where *f*_OC_ is the organic carbon fraction, *f*_c_ the clay fraction, and *f*_S_ the silt fraction.

### Soil physical properties

Setting up appropriate values for the soil physical parameters is very important, because they govern the hydraulic behaviour of the soils and their interactions with plants. In APSIM *Classic* the main values are entered in the Water node, with texture and rocks specified in the Analysis node. In APSIM *Next Generation* all the values are set up in the Physical node. Some additional parameters, specific to the water balance model in use, are entered in the dedicated model node (i.e., SoilWater or SWIM3).

#### Particle size distribution

S-map provides information on the particle size classes of sand, silt, and clay. As the limit between sand and silt for NZ is 0.06 mm, and in APSIM the limit is based on the International system with 0.05 mm, a correction should ideally be made [26,27]. It is common practice to use these texture categories to characterise soils. Therefore, the thresholds between the particle size classes need to be consistent, especially when they are used to obtain other parameter values based on pedotransfer functions. In APSIM, the clay content is used for the SoilTemperature model. Although the sand and silt percentages are not currently used, they should be entered for consistency.

#### Rock fragment content

The fraction (percentage) of coarse fragments (≥ 2.0 mm) for each functional horizon of the soil is provided by S-map. Note that the parameter values in the Physical node are adjusted according to the percentage of rocks in each soil layer, using a modified version of Bouwer and Rice 1984:

$$P_{adj}=P_{matrix}\left(1-f_{rocks}\right)+\;f_{rocks\;}P_{rocks}$$

where $f_{\mathrm{rocks}}$ is the fraction of rocks in each layer, $P_{matrix}$ is the soil water parameter for the soil matrix and $P_{rocks}$ is the equivalent parameter for the rock fraction. The equation assumes that the presence of rocks reduces the volume fraction of the hydraulicly active soil linearly. Note that the correction equation above is not applied to hydraulic conductivity, which is assumed to be entered for the intact soil.

#### Bulk density and particle density

The value of the bulk density (BD) for each functional horizon is estimated from S-map information on the soil layer's total porosity and particle density. The algorithm for calculating BD is based on the particle density (PD):

BD=PD(1−TP)

where TP is the total porosity PD the particle density. The latter is dependent on the soil order and texture: for the soil orders M and L the particle density is set to 2.45; for G, Z and N the particle density is set to 2.53; and for R, P, B, A, W, E, S, and X the particle density is set to 2.65. If the functional horizon contains >80% sand and <8% clay, the particle density is set to 2.8. Note that BD is also adjusted to give the density of fine (<2.0 mm) particles in the soil volume if *f*_Rocks_ is >0:

$$BD_{adj}=BD_{matrix}\left(1-f_{rocks}\right)+\;f_{rocks\;}BD_{rocks}$$

where $f_{\mathrm{rocks}}$ is the fraction of rocks in each layer, $BD_{matrix}$ is the bulk density for the soil matrix, and $BD_{rocks}$ is the equivalent parameter for the rock fraction.

#### Soil water retention values: AirDry, LL15, DUL, and SAT

The water characteristics of each layer of soil are specified in terms of three thresholds [12]: the lower limit (LL15), the drained upper limit (DUL), and the saturated (SAT) volumetric water content (cm^3^ cm^−3^), plus a value for the air dry (AirDry) volumetric water contents. The values of LL15, DUL, and SAT come directly from S-map, and are based on a pedotransfer function (Ptf), which predicts the soil water for various tensions based on soil order, rock class, functional horizon characteristics, and depth, as well as the estimated texture. The Ptf is described in McNeill, Lilburne [28], but has been updated in 2020 to better represent some soil orders. AirDry represents the soil moisture reached after evaporation (cm^3^ cm^−3^). For functional horizons above 300 mm, the AirDry value is set to half that of LL15. Below 300 mm, the AirDry value is set equal to that of LL15. If a functional horizon extends across the 300 mm boundary, a weighted mean of the air-dry values of the two adjacent functional horizons is used. The LL15 field represents the soil moisture values for the lower limit of water movement, often termed the permanent wilting point, and defined at –15 bar (1.5 MPa). The drained upper limit (DUL) represents the soil water content above which drainage due to gravity starts, and also the upper limit for the definition of plant-available water. It is also known as ‘field capacity’, and is generally assigned to a water potential of –0.1 bar (10 kPa).

SAT is calculated from the TP for each layer, assuming that SAT is 93% of the total porosity. Note that the total porosity is adjusted by the proportion of rocks in the functional horizon.

In some cases the estimation of SAT may be less than the estimate of DUL from the S-map Ptf, in which case the following adjustments should be done for a given layer:

SAT=DUL+0.75(TP−DUL)forDUL≥0.93TP

#### Saturated hydraulic conductivity

S-map provides *K*_S_ (denoted fhKsat) in mm hr^−1^, and for APSIM the value needs to be converted to mm d^−1^. The *K*_S_ values in S-map are based on measured values for different topsoils and subsoils from the S-map database, which have been used to assign *K*_S_ values to functional horizons (values are provided in the supplementary material, Table S1). When using SoilWater the value of $K_{S}$ is optional. If a value is provided, $K_{S}$ limits infiltration at saturation and induces surface ponding; otherwise any water above saturation either runs off or drains to a deeper layer that has capacity.

### Soil–plant-specific parameters

Although in APSIM plant and soil processes are simulated with separate models, a few parameters are needed to account for the interactions between the soil and the plant. Three parameters (LL, KL, and XF) need to be provided in the soil node for each layer, up to the maximum rooting depth, $RZ_{max}$. Because these parameters control the plant–soil interaction, their value is zero below $RZ_{max}$. $RZ_{max}\;$depends on the crop, the cultivar, and the growing conditions. Values for a selection of crops are provided by S-map, and these are based on information from the Foundation for Arable Research [29] and expert opinion (Table 1).

**Table 1:** Soil–plant-specific factors provided by S-map for a selection of crops, with KL_0_ = KL at the surface (mm mm^−1^ d^−1^). $RZ_{max}$ = maximum rooting depth (mm), PR_resp_ = PRresponse) and PR_thresh_ = PRthreshold AgPasture is a module for simulating mixed pastures of C3 and C4 grasses, as well as legumes [30], and SCRUM a **S**imple **C**rop **R**esource **U**ptake **M**odel [31].

Crop	Kl_0_	$RZ_{max}$	PR_resp_	PR_thresh_
Barley	0.06	1500	1.20	2
Canola	0.06	1200	0.96	0.8
Clover seed	0.06	900	1.04	1.2
Fieldpea	0.06	900	0.80	0
Fababean	0.06	1200	0.96	0.8
Kale	0.06	3000	0.96	0.8
Italian ryegrass	0.06	700	0.96	0.8
Lucerne	0.05	3000	1.20	2
Maize	0.06	1500	0.96	0.8
Oats	0.06	1000	1.04	1.2
Potato	0.06	1000	0.80	0
Grass seed	0.06	900	0.96	0.8
Triticale	0.06	1500	1.04	1.2
Wheat	0.06	1500	1.04	1.2
Ryegrass/clover	0.1	800	0.96	0.8
Ryegrass	0.1	700	0.96	0.8
White clover	0.1	300	0.80	0
AgPasture	0.1	900	0.96	0.8
SCRUM	0.06	1500	1.04	1.2

The values of LL (m^3^ m^−3^) define the lower limit for water extraction by plants, and should be set equal to LL15 for most agricultural crops.

KL is an empirical factor (mm water uptake per mm water stored in the soil layer per day) and represents the maximum fraction of the available water a crop can take up daily from each soil layer. Thus it combines the effects of soil hydraulic properties (e.g., water conductance) and plant root characteristics (e.g., root length density and activity). The value of KL, when based on information from S-map, is calculated from a depth-dependent crop potential (CP), a depth factor *f*(z), and a functional-horizon-specific KL factor (KL_fh_), which characterises the effect of each functional horizon type on the distribution of roots and the rate of conductance of water to roots:

$$\text{KL}=\text{CP}\;KL_{fh}$$

The crop potential for each layer is calculated as:

$$\text{CP}=KL_{0}\;f\left(z\right)$$

with

$$f\left(z\right)=1\;\text{for}\;\bar{z\;}\;\leq 300\;mm$$

$$f\left(z\right)=\exp\left[-0.002\;\left(\frac{1500}{RZ_{max}-300}\right)\left(\bar{z}-300\right)\right]\;\text{for}\;\bar{z\;}>300\;mm$$

Values for KL_fh_ are provided in the Supplementary Material (Table S1).

The value of XF, dimensionless with values 0–1, is the root exploration factor, a simple empirical factor that limits root growth in any soil layer due to physical (i.e., compacted layer, low oxygen status, fragipan, water table) or chemical (i.e. salinity) constraints. The value is also based on the crop and the functional horizon, because the attributes of a functional horizon affect the rate of root penetration into the soil. Very slow root penetration was assigned to dense, massive soils and extremely stony soils that lack fines. Soils with free root penetration included most soils from pumice, volcanic ash or peat, and loamy soils with weak consistency. The remaining soils were ranked between these groups mainly according to packing density.

The estimation of XF in S-map is based on an algorithm that accounts for resistance. It is based on three crop-based parameters: PResistance (PR_resist_), PRresponse (PR_resp_) and PRthreshold (PR_thresh_) with:

$$\text{XF}=\;1\;\text{if}\;\;PR_{resist}\;<PR_{thresh}$$

$$\text{XF}=\exp\left[\;PR_{resp}\;\left(\;PR_{resist}-PR_{thresh}\right)\right]\;\text{if}\;\;PR_{resist}\;>PR_{thresh}$$

with

$$PR_{resist}=\;XF_{fh}\;x\;5$$

Values for XF_fh_ are provided in the Supplementary Material (Table S1).

### Soil organic matter

Soil organic matter is described in APSIM as conceptual pools (Probert *et al.*, 1998). The initial vales for these, which include both C and N, are entered in the SoilOrganicMatter node in APSIM *Classic* version, and in the Organic node in APSIM *Next Generation*. The required parameters include those for fresh organic material (FOM) and for soil organic matter (SOM). Only the SOM set-up based on S-map is described here; for FOM, see Cichota et *al.,* submitted)

Organic matter is set up by providing the content of organic carbon (OC, %) for each layer, and a corresponding C:N ratio to define the amounts of N in each soil layer. In APSIM *Classic*, C:N is assumed to be constant over the soil layers. Furthermore, values for FBIOM (fraction of OM with a fast turnover rate, with a C:N of 8 commonly used), and FINert (inert fraction) need to be provided.

S-map provides values for OC, FBIOM, and FINert, which depend on the previous land use (prior to the start of the simulation period) and the mean depth $\bar{z}$ (cm) of each layer.

If the previous land use is ‘Pasture’, the values are calculated as:

$$\text{OC}=4.7\;\text{for}\;\bar{z}\leq 15$$

$$\text{OC}=0.2+4.5\;e^{\left(-0.08\;\bar{z}-15\right)\;}\text{for}\;\bar{z}>15$$

$$\text{FBIOM}=0.01+0.08\;e^{\left(-0.06\;\bar{z}\right)}$$

$$\text{FINert}=0.5+0.5\;\left(1-e^{\left(-0.03\;\bar{z}\right)}\right)$$

If the previous land use is ‘Crop’, the values are calculated as:

$$\text{OC}=2.6\;\text{for}\;\bar{z}\leq 15$$

$$\text{OC}=0.2+2.4\;e^{\left(-0.08\;\bar{z}-15\right)\;}\text{for}\;\bar{z}>15$$

$$\text{FBIOM}=0.01+0.04\;e^{\left(-0.06\;\bar{z}\right)}$$

$$\text{FINert}=0.15+0.85\;\left(1-e^{\left(-0.03\;\bar{z}\right)}\right)$$

### Model-specific parameters

Some child nodes within the soil node in APSIM correspond to specific models. When SoilWater is used, a SoilWater node is present, and when SWIM3 is used, a Swim3 node is present, which also requires an additional child node, SoluteParameters. These nodes require various parameters for simulating runoff, evaporation, and water movement. Here only the values for SWCON for SoilWat and for K_DUL_ (SWIM3) are discussed. For the others see Cichota et al. (submitted).

SWCON specifies the proportion of water near saturation (above DUL) that will drain per day from each soil layer. The value is thus, in theory, linked to hydraulic conductivity as well as water storage and layer thickness. This value is supplied by S-map based on the functional horizon (supplementary material), and is currently independent of the layer thickness. Values specific to functional horizons for SWCON were derived by comparing simulated drainage rates using SWIM3 with those obtained using SoilWater. For this, soil profiles were set up with a range of attributes that affect the unsaturated drainage rate (texture, rock fragment content, structure size) for a range of functional horizons. The value that was closest to the drainage rate based on SWIM3 was then assigned to the functional horizon. SWCON values were then assigned to the remaining functional horizons based on the relationships of other functional horizon attributes to the modelled functional horizons (values are provided in the Supplementary Material, Table S1). A sensitivity analysis on the value of SWCON, and the effect of layer thickness, is provided below.

Swim3 requires a value for *K*_DUL_, which is the hydraulic conductivity at the drained upper limit tension (taken as –10 kPa). A single value is used for the whole soil profile. A K_DUL_ value of about 0.1 mm day^−1^ is typically used in the literature [32,33]. Based on a sensitivity analysis and expected ephemeral soil saturation levels and morphology, Vogeler, Carrick [34] suggested that for well-drained soils in NZ, *K*_DUL_ ranges from 1 to 5 mm d^−1^ should be used, for moderately well-drained soils from 0.1 to 0.5 mm d^−1^, and for poorly drained soils values from 0.05 to 0.1 mm d^−1^.

In the SoluteParameters child node, parameters of the Freundlich isotherm need to be specified for the solutes typically simulated in APSIM. Only the values for NH_4_ are specified by default, with other solutes being given values for no adsorption. Users can alter these if desired in a particular simulated system. The Freundlich isotherm is given by:

$$S=k\;C^{n}$$

where *S* is the concentration of an ion adsorbed to soil particles (mg kg^−1^ soil), *C* the concentration in the soil solution (mg m^−3^), and *k* (m^3^ kg^−1^) and *n* (-) are Freundlich fitting parameters, termed *Exco* and *Fip* in APSIM. S-map uses the following equations to calculate these parameters, based on measurements of NH_4_ adsorption for New Zealand soils [35]:

$$k=30.841-1.263\;\text{CEC}+1.235\;f_{clay}$$

$$n=0.46+0.013\;\text{CEC}-0.006\;f_{clay}$$

where *CEC* is the cation exchange capacity (cmol^+^ kg^−1^) and *f*_c_ is the clay fraction (%).

## Method evaluation

### Effect of soil layer thickness and SWCON on water movement and storage

The effect of the setup of the soil layering was investigated based on two sets of modelling experiments, both based on data from the Waikato region. The first was a lysimeter experiment in which drainage was measured through a series of different soils and with different irrigation regimes. For the study here only the lysimeters with the well-drained Horotiu silt loam, receiving a total water input of 1100 mm year^−1^ (rainfall plus irrigation) were used. The lysimeters were 70 cm in depth, contained a ryegrass/white clover mixture, and received a urine application equivalent to 1000 kg N ha^−1^ in May 2008. Further details are provided in Shepherd *et al.*, 2009.

The second set of experiments was carried out to measure N_2_O emissions from animal excreta, in which soil water content in the top 75 mm was measured gravimetrically by taking soil samples (75mm deep, 25mm diameter) throughout the experiments. Only the control plots without excreta were used here, and only those done on the Horotiu soil. The data series consisted of seven different experiments, each done on a different site, between 2000 and 2009. Details of the experiments are provided in Giltrap, Vogeler [36].

For both experiments, APSIM was used to simulate water flow using SoilWater, with soil descriptions either based on laboratory measurements from a site on the same generic soil type as the experimental site (Horotiu), or on a sibling created in S-map to match the site description. Note that S-map uses the updated soil type name, Otorohanga (Otor_70a.1). Key soil properties as set up for the APSIM simulations are provided in the Supplementary Material (Table S2). Thus, the former uses measurements from a representative soil, the latter uses Ptf-estimated values based on a soil description of the experimental site. The effect of different layering was also investigated. For the Horotiu soil, two different depth layering scenarios based on the measured soil profile were used: the first (Layering 1) was more coarse, with a top layer of 20 cm, and the remaining layers were 40 cm (thus for the lysimeters: 0–20; 20–60; 60–70 cm); the second layering (Layering 2) was finer, as recommended by Moore, Holzworth [37], with depth increments of 15 cm (thus 0–15; 15–30; 30–45; 45–60; 60–70). Furthermore, for SWCON, either the value recommended by Moore, Holzworth [37] for a silt soil of 0.5 was used for each of the layers, or SWCON was calculated for each layer based on the hydraulic properties of the soil and the layer thickness, following the approach taken by [38]. To illustrate the effect of SWCON, values of 0.3 (as recommended for clay soils) and of 0.7 (as recommended for sandy soils) were also compared. For the Otor_70a.1 soil, three different depth-layering scenarios were used: the first (Layering 1) was more coarse, and lumped horizons with similar texture and bulk density into one functional horizon (0–31; 31–55; 55–70); the second had a finer resolution at the top (0–6; 6–31; 31–55; 55–70); and the third had an even finer resolution in horizons near the surface (0–6; 6–17; 17–31; 31–61; 61–70).

**Fig. 1:**
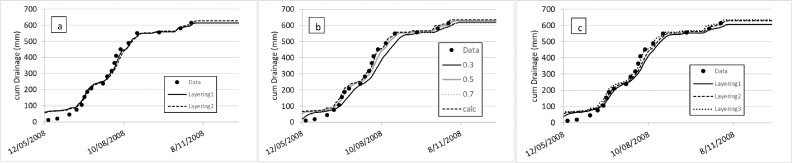
Measured and simulated cumulative drainage from lysimeters (with depths of 70 cm) under a ryegrass/white clover pasture, with a representative generic soil description of a Horotiu silt loam, with (a) showing the effect of layering with SWCON of 0.5 (Layering 1: 0–20 cm, 20–60 cm, 60–70 cm and Layering 2: 0–15 cm, 15–30 cm, 30–45 cm, 45–60 cm, 60–70 cm), and (b) the effect of SWCON, either set to different values (0.3, 0.5 or 0.7) or calculated based on hydraulic properties and layer thickness, using Layering 2. Simulations in c) are based on S-map descriptions (Otor_70a.1) with different layering (Layering1: 0–31 cm, 31–55 cm, 55–70 cm, Layering 2: 0–6 cm, 6–31 cm, 31–55 cm, 55–70 cm, and Layering 3: 0–6 cm, 6–17 cm, 17–31 cm, 31–61 cm, 61–70 cm)

The simulations show that, based on the representative Horotiu soil description, neither the layering nor SWCON had much effect on the amount and timing of drainage from the lysimeter. Only the slowest SWCON value of 0.3 shows a slightly delayed drainage (Fig. 1). Based on the S-map Otor_70a.1 soil description, simulations are also similar for the three layering scenarios, with the finer layering at the surface showing slightly better prediction of the drainage.

For the second set of experiments, the soil attribute parametrisation from the representative generic soil description of the Horotiu soil was tested for predicting soil water content dynamics across a suite of seven field plots. The simulation results indicate little effect of the layering, either coarse (Layering 1) or finer (Layering 2). The use of a texture-based SWCON (i.e. the S-map estimate) compared to values calculated based on representative soil hydraulic properties and the layer thickness also showed little effect, with very similar statistical goodness of fit values compared with measured soil water content (Table 2). When the soil description was taken from S-map, the best performance was obtained for coarser layering at the top (Layering 1), and this provided better predictions than the simulations with any of the measured generic Horotiu soil-layering scenarios.

**Table 2:** Model performance statistics for the prediction of temporal soil water contents at a depth from 0 to 75 mm from a series of field experiments done on the Horotiu soil in the Waikato region of New Zealand with APSIM-SoilWat, with either a description of a representative generic Horotiu soil or S-map. Different layering, and different values for SWCON were used, either based on texture (0.5) or calculated based on hydraulic properties and layer thickness according to Suleiman and Ritchie (2004). corr = correlation (-); NSE = Nash Sutcliffe efficiency score (-); RMSE = root mean squared error (m^3^ m^−3^). For the Horotiu soil the layering was: Layering1: 0–20 cm, 20–60 cm, 60–70 cm and Layering 2: 0–15 cm, 15–30 cm, 30–45 cm, 45–60 cm, 60–70 cm, and for S-map descriptions Layering1: 0–31 cm, 31–55 cm, 55–70 cm, Layering 2: 0–6 cm, 6–31 cm, 31–55 cm, 55–70 cm, and Layering 3: 0–6 cm, 6–17 cm, 17–31 cm, 31–61 cm, 61–70 cm).

		corr	NSE	RMSE
Horotiu		SWCON = 0.5		
	Layering1	0.64	0.12	0.072
	Layering2	0.64	0.10	0.072
		SWCON = calculated		
	Layering1	0.66	0.04	0.075
	Layering2	0.63	0.13	0.071
S-map		SWCON = 0.3		
	Layering1	0.59	0.28	0.065
	Layering2	0.63	–0.11	0.080
	Layering3	0.59	–0.27	0.086

**Fig. 2:**
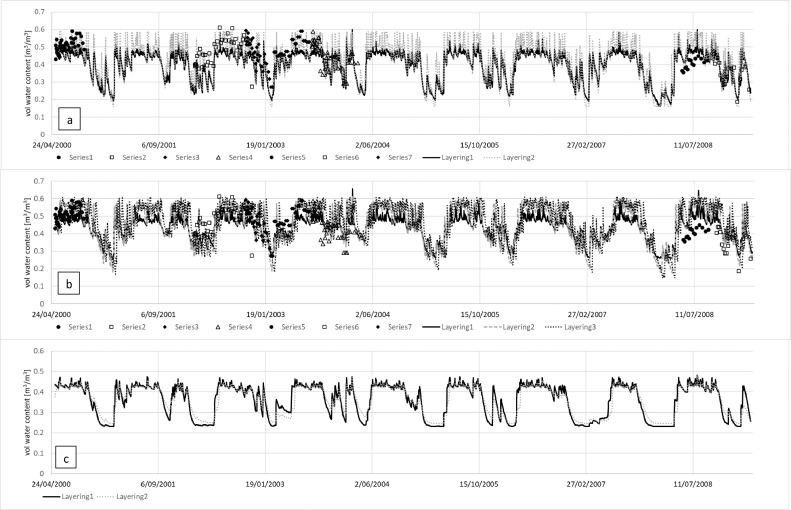
Measured and APSIM simulated soil water content from seven different sites and periods (labelled Series 1 to 7) of field experiments done on the Horotiu soil in the Waikato region of New Zealand. APSIM simulations were done with soil descriptions based on representative generic descriptions of the Horotiu soil (a and c) and S-map (b). Measurements were done at a depth from 0–75 mm (a and b), and from 20–60 cm (c). For the Horotiu soil the layering was Layering1: 0–20 cm, 20–60 cm, 60–70 cm and Layering 2: 0–15 cm, 15–30 cm, 30–45 cm, 45–60 cm, 60–70 cm; and for S-map descriptions Layering1: 0–31 cm, 31–55 cm, 55–70 cm, Layering 2: 0–6 cm, 6–31 cm, 31–55 cm, 55–70 cm, and Layering 3: 0–6 cm, 6–17 cm, 17–31 cm, 31–61 cm, 61–70 cm).

While the layering affects the prediction of the soil moisture in the topsoil (Fig. 2), with Horotiu and Layering 2 generally having higher soil moisture content throughout the measurement periods, some of the experimental data are better described with Layering1 and some with Layering 2. The layering had only a small effect on the prediction of the temporal soil moisture at a depth from 20 to 60 cm. Similarly, simulations with S-map show under- and over-prediction of the soil moisture for the various measurement periods. Site-specific soil data for the seven experiments were not measured, which probably explains the fact that the calculated SWCON based on hydraulic properties only showed slightly better goodness of fit values. Such measurements are important when processes highly sensitive to soil moisture, such as denitrification and N_2_O emissions, are simulated [39]. If accurate estimates of denitrification and N_2_O emissions are required, it might be advisable to obtain site-specific soil hydraulic measurements.

### Effect of SOM pools on N leaching and plant growth

Here the effect of the varying input parameters associated with the setup, size, and distribution of SOM pools on outcomes such as plant growth and N leaching was investigated. Initially, the first experiment described in section 5.1 was simulated, whereby irrigated pasture lysimeters containing well-drained Horotiu silt loam received a urine application equivalent to 1000 kg N ha^−1^ [40]. As above, the soil description was based on a representative generic Horotiu soil. This represented a scenario where there was excess mineral N in the system for plant growth and substantial N losses through leaching. The same soil and climate were then used to simulate a rain-fed winter wheat – fallow – winter wheat rotation, sown in autumn, harvested in summer, with 30 kg N ha^−1^ mineral fertiliser applied at sowing and a further 150 kg N ha^−1^ in spring. This represented a scenario where there was adequate mineral N in the system for plant growth but unlikely to be substantial N losses through leaching.

For both scenarios, APSIM 7.10 was used to simulate dry matter (DM) production and N leaching using SoilWat with a 1-year spin-up to allow the soil and plant variables to adjust to environmental conditions, thus reducing the effects of the initial conditions. These scenarios were then run without the 1-year spin-up phase and the S-map soil description (Otor_72a.1). Variation in the input values for OC, FBIOM, and FINert were also investigated to reflect variation in measurements and uncertainty relating to appropriate parameterisation of conceptual carbon pools. OC was increased and decreased by 20%, FBIOM was doubled and halved, and FINert was increased and decreased by 20% to a depth of 20 cm, 10% from 20 to 60 cm, and 1% below 60 cm.

The simulations showed that in the pasture scenario, DM production was not substantially affected by the absence of a spin-up phase, changing the whole soil description to that derived from S-Map or variations in input parameters for SOM (Table 3). The greatest difference in DM production from the base scenario was 0.4 t ha^−1^ or 3%. This is probably due to there being excess N within the system resulting from the urine patch, and as such adequate mineral N for plant growth. The same is also true for nitrate leaching, with the greatest difference being 8 kg ha^−1^ or 1.5%. However, when the S-map soil description was used, the difference in N leaching was -442 kg ha^−1^ or –83%. While there were differences in input parameters for SOM pools, there were also substantial differences in soil input parameters that influence the storage and movement of water in the soil in the S-map soil description compared to the representative generic Horotiu soil description, which would have a substantial impact on predictions of nitrate leaching. This further reinforces that for very site-specific research questions, the use of site-specific parameters is important and can substantially affect simulated outcomes.

**Table 3:** Simulated dry matter production and nitrate leaching under pasture (with a urine patch, equivalent to 1000 kg N ha^−1^) and winter wheat scenarios. Values are given for (i) a base scenario that used a representative generic Horotiu soil description with a spin-up phase, (ii) removal of the spin-up phase, (iii) replacement if the soil description with S-map, and (iv) variations in OC, FBiom, and FInert, where OC was increased and decreased by 20%, FBiom was doubled and halved, and FInert was increased and decreased by 20% to a depth of 20 cm, by 10% from 20 to 60 cm, and by 1% below 60 cm.

Scenario		Pasture (with urine patch)				Winter wheat			
		Values		Diff. from base		Values		Diff. from base	
		Dry matter (t ha^−1^)	Nitrate leaching (kg ha^−1^)	Dry matter (t ha^−1^)	Nitrate leaching (kg ha^−1^)	Dry matter (t ha^−1^)	Nitrate leaching (kg ha^−1^)	Dry matter (t ha^−1^)	Nitrate leaching (kg ha^−1^)
Base		13.9	534.4	–	–	16.1	36.3	–	–
No spin up		14.8	526.4	0.9	–8.0	14.5	27.5	–1.6	–8.8
S–map		13.6	92.8	–0.3	–441.6	14.6	20.0	–1.4	–16.3
OC	20% more	14.0	530.3	0.1	–4.1	16.6	43.4	0.6	7.1
	20% less	13.7	539.2	–0.2	4.8	15.3	30.3	–0.7	–6.0
Fbiom	Double	14.3	536.6	0.4	2.2	17.3	62.3	1.2	26.0
	Half	13.5	533.7	–0.3	–0.7	14.5	25.6	–1.5	–10.7
Finert	More	13.5	532.8	–0.4	–1.6	14.3	24.5	–1.7	–11.8
	Less	14.1	536.4	0.3	2.0	17.2	51.4	1.1	15.1

Varying the input parameters in the winter wheat – fallow – winter wheat rotation had a greater impact on both DM production and nitrate leaching (Table 3). In the no-spin-up scenario, compared to the base scenario (with a 1-year spin-up) there was a 10% reduction in DM production and a 24% reduction in nitrate leaching. This is due to the initialisation of the SOM pools not being at equilibrium for the system in question. With a longer spin-up period, differences in simulated outcomes would probably be greater. In the S-map scenario, compared to the base scenario that used soil representative generic measured values, there was a 9% reduction in DM production and a 45% reduction in nitrate leaching. Again, this indicates the likely importance of site-specific measured data.

In the same winter wheat – fallow – winter wheat rotation system, when there were changes to the initialisation parameters that increased the amount of active SOM in either the HUM or BIOM pools (i.e., increasing OC or FBIOM, or decreasing FINert) there was an increase in both DM production and nitrate leaching compared to the base scenario. Differences in values of simulated DM production and nitrate leaching ranged from 0.6 t ha^−1^ and 7 kg ha^−1^, respectively, with a 20% increase in OC, to 1.2 t ha^−1^ and 26 kg ha^−1^, respectively, when FBIOM was doubled. The reverse was true for decreasing the amount of active SOM, with a decrease in those simulated outcomes. Differences in values of simulated DM production and nitrate leaching ranged from –0.7 t ha^−1^ and –6 kg ha^−1^, respectively, with a 20% decrease in OC, to –1.7 t ha^−1^ and -12 kg ha^−1^, respectively, when FBIOM was doubled. This was due to those parameters influencing the total amount of decomposition as the size of those active pools changed, which resulted in changes in N mineralisation and different amounts of mineral N available in the system. While the same occurred in the pasture scenario, in the winter wheat scenario these parameters had a larger impact on simulation outcomes, because there were only adequate amounts of mineral N in the system.

This analysis emphasises the need for a spin-up period at the beginning of the simulation when modelling mineralisation and/or N leaching (unless there is ample N in the system, as in our example of the urine patch modelling), the importance of site-specific measured soil data, and the appropriate parameterisation of the SOM pools (Cichota *et al.,* submitted).

### Effect of KDUL and Ksat on modelling temporal soil moisture content

Through inverse modelling using APSIM with SWIM3, Vogeler, Carrick [41] found that in soils with a slowly permeable subsurface horizon the value of K_sat_ only had a small effect on temporal soil moisture contents and level of saturation, whereas the K_DUL_ was much more important. Based on a sensitivity analysis, and expected ephemeral soil saturation levels from the soil morphology, Vogeler, Carrick [34] then suggested that for well-drained soils in NZ, *K*_DUL_ ranges from 1 to 5 mm d^−1^, for moderately well drained soils from 0.1 to 0.5 mm d^−1^, and for poorly drained soils from 0.05 to 0.1 mm d^−1^. With the default value for *K*_DUL_ of 0.1 mm d^−1^, the saturation level would be overestimated in well-drained soils and underestimated in poorly drained soils. Although, in this study the effect of poor prediction of the saturation level on denitrification and potential nitrous oxide emissions was not shown, these losses would be likewise over- and underestimated, given a sufficient amount of N in the soil. Furthermore, the choice of *K*_DUL_ affected pasture yield, with both reduced yield (up to 13%) and increased yield (up to 8%), depending on the soil and climatic condition.

### Effect of the choice of KL

Based on a sensitivity analysis, [42] showed the effect of KL and the decay rate of KL with depth, *λ*_KL_ (equivalent to *f*(z)), on soil water content and crop uptake. From temporal soil measurements to a depth of 1.5 m they inferred that simulations of soil water content were most sensitive to KL of the upper most soil layer, followed by the decay rate of KL with depth (*λ*kl). For both lucerne and ryegrass, a KL value of 0.11 d^−1^ provided the best fit, whereas *λ*kl differed among species, being higher for shallow fibrous ryegrass roots (–0.01) than for the deep lucerne taproots (–0.002). While this approach, using detailed field measurements, sensitivity analysis, and optimisation, is valid for defining crop-soil-specific parameters, so far this has only been done for one soil type and two crops. Further work is needed to derive these crop-soil specific parameters for the various functional horizons, as well as testing of the approach compared with the current one used in S-map (see section 4.5).

## Conclusions

The paper provides a detailed protocol for how to use soil information from S-map as input parameters for APSIM. Furthermore, the sensitivity of various parameters to water movement, water storage, and N cycling was investigated. The analysis showed that for regional or national assessments the use of S-map information is appropriate and provides similar results compared to soil descriptions based on layer-specific measurements from a representative generic soil. Existing and ongoing high-resolution soil characterisation efforts for experimental and modelling research aimed at improving our understanding of soil processes and modelling approaches are essential.

## Declaration of Competing Interest

The authors confirm that there are no conflict of interest

## Appendix Supplementary materials

Image, application 1

Image, application 2
