# Supplementary material for: S-map parameters for APSIM
Source: MethodsX. 2022 Feb 6;9:101632. doi: 10.1016/j.mex.2022.101632 (PMC8861821; doi:10.1016/j.mex.2022.101632)
Supplement: Supplementary file 1 [file mmc1.docx]

Table S1. Functional horizons (FH) from S-map, and functional horizon specific values of SWCON (-), saturated hydraulic conductivity (Ks; mm hr^-1^), the maximum fraction of the available water, which a crop can take up daily from each soil layer (KLfh; mm water uptake /mm water stored in the soil layer /day), and the root exploration factor (XFfh; 0-1)

| FH | SWCON | Ks (mm/hr) | KL_fh_ | XF_fh_ |
| --- | --- | --- | --- | --- |
| Aa | 0.6 | 250 | 0.15 | 0.5 |
| Af | 0.4 | 40 | 0.25 | 0.4 |
| Al | 0.5 | 150 | 0.35 | 0.2 |
| As | 0.45 | 50 | 0.3 | 0.2 |
| Aw | 0.45 | 80 | 0.35 | 0.1 |
| LCf | 0.15 | 1 | 0.15 | 0.6 |
| LCs | 0.2 | 8 | 0.25 | 0.3 |
| LFf | 0.2 | 5 | 0.15 | 0.4 |
| LFs | 0.3 | 20 | 0.25 | 0.2 |
| Lw | 0.3 | 40 | 0.3 | 0.05 |
| tAa | 0.6 | 250 | 0.2 | 0.3 |
| tAf | 0.4 | 60 | 0.25 | 0.2 |
| tAl | 0.5 | 200 | 0.3 | 0.15 |
| tAs | 0.5 | 80 | 0.3 | 0.1 |
| tAw | 0.5 | 150 | 0.3 | 0.05 |
| tLCf | 0.25 | 10 | 0.15 | 0.35 |
| tLCs | 0.3 | 30 | 0.25 | 0.2 |
| tLFf | 0.3 | 30 | 0.2 | 0.25 |
| tLFs | 0.35 | 40 | 0.3 | 0.1 |
| tLw | 0.3 | 60 | 0.3 | 0.05 |
| tYC | 0.25 | 10 | 0.2 | 0.25 |
| tYFf | 0.3 | 30 | 0.2 | 0.25 |
| tYFs | 0.3 | 40 | 0.3 | 0.2 |
| tYFws | 0.3 | 40 | 0.3 | 0.2 |
| tYw | 0.35 | 40 | 0.3 | 0.1 |
| YC | 0.15 | 3 | 0.12 | 0.4 |
| YFf | 0.2 | 5 | 0.15 | 0.35 |
| YFs | 0.25 | 15 | 0.25 | 0.2 |
| Yw | 0.3 | 15 | 0.3 | 0.15 |
| SAf | 0.4 | 40 | 0.25 | 0.4 |
| SAl | 0.5 | 150 | 0.35 | 0.2 |
| SAs | 0.45 | 50 | 0.3 | 0.2 |
| SAw | 0.5 | 80 | 0.35 | 0.1 |
| SLCf | 0.15 | 1 | 0.15 | 0.6 |
| SLCs | 0.2 | 8 | 0.25 | 0.3 |
| SLFf | 0.2 | 5 | 0.15 | 0.4 |
| SLFs | 0.3 | 20 | 0.25 | 0.2 |
| SLw | 0.35 | 40 | 0.3 | 0.05 |
| SYC | 0.15 | 3 | 0.12 | 0.4 |
| SYFf | 0.2 | 5 | 0.15 | 0.35 |
| SYFs | 0.25 | 15 | 0.25 | 0.2 |
| SYFws | 0.25 | 15 | 0.25 | 0.2 |
| SYw | 0.3 | 15 | 0.3 | 0.15 |
| tSAf | 0.4 | 60 | 0.25 | 0.2 |
| tSAl | 0.5 | 200 | 0.35 | 0.15 |
| tSAs | 0.5 | 80 | 0.3 | 0.1 |
| tSAw | 0.5 | 150 | 0.3 | 0.05 |
| tSLCf | 0.25 | 10 | 0.15 | 0.35 |
| tSLCs | 0.3 | 30 | 0.25 | 0.2 |
| tSLFf | 0.25 | 30 | 0.2 | 0.25 |
| tSLFs | 0.35 | 40 | 0.3 | 0.1 |
| tSLw | 0.3 | 60 | 0.3 | 0.05 |
| tSYC | 0.25 | 10 | 0.2 | 0.25 |
| tSYFf | 0.25 | 30 | 0.2 | 0.25 |
| tSYFs | 0.3 | 40 | 0.3 | 0.2 |
| tSYFws | 0.3 | 40 | 0.3 | 0.2 |
| tSYw | 0.35 | 40 | 0.3 | 0.15 |
| tVAl | 0.5 | 200 | 0.35 | 0.15 |
| tVLc | 0.3 | 40 | 0.2 | 0.2 |
| tVLd | 0.2 | 4 | 0.15 | 0.4 |
| tVLl | 0.3 | 60 | 0.3 | 0.05 |
| tVYc | 0.2 | 40 | 0.25 | 0.2 |
| tVYd | 0.2 | 4 | 0.15 | 0.3 |
| tVYl | 0.3 | 40 | 0.3 | 0.15 |
| tXA | 0.5 | 200 | 0.35 | 0.2 |
| tXL | 0.3 | 60 | 0.3 | 0.05 |
| VAc | 0.4 | 50 | 0.25 | 0.3 |
| VAd | 0.2 | 20 | 0.1 | 0.6 |
| VAl | 0.5 | 100 | 0.4 | 0.25 |
| VLc | 0.2 | 8 | 0.2 | 0.3 |
| VLd | 0.15 | 0.05 | 0.1 | 0.75 |
| VLl | 0.35 | 40 | 0.3 | 0.05 |
| VYc | 0.2 | 15 | 0.2 | 0.25 |
| VYd | 0.15 | 1 | 0.1 | 0.6 |
| VYl | 0.3 | 20 | 0.3 | 0.15 |
| XA | 0.5 | 150 | 0.4 | 0.3 |
| XL | 0.3 | 40 | 0.3 | 0.2 |
| Xx | 0.6 | 250 | 0.15 | 0.6 |
| XY | 0.2 | 15 | 0.25 | 0.2 |
| LEw | 0.3 | 40 | 0.3 | 0.05 |
| YFws | 0.25 | 15 | 0.3 | 0.2 |
| tJ | 0.3 | 10 | 0.3 | 0.2 |
| tLEw | 0.3 | 60 | 0.3 | 0.05 |
| J | 0.3 | 10 | 0.3 | 0.5 |
| Q | 0.05 | 0.01 | 0 | 1 |
| LK | 0.1 | 1 | 0.1 | 0.6 |
| Oh | 0.3 | 50 | 0.4 | 0.1 |
| OhA | 0.3 | 60 | 0.4 | 0.15 |
| OhL | 0.3 | 20 | 0.4 | 0.1 |
| OhzA | 0.3 | 60 | 0.4 | 0.15 |
| OhzL | 0.3 | 20 | 0.4 | 0.1 |
| Or | 0.05 | 100 | 0.3 | 0.15 |
| tOh | 0.3 | 80 | 0.4 | 0.05 |
| tOhA | 0.45 | 80 | 0.4 | 0.2 |
| tOhL | 0.3 | 50 | 0.4 | 0.1 |
| tOhY | 0.3 | 50 | 0.4 | 0.1 |
| tOhzA | 0.3 | 100 | 0.4 | 0.2 |
| tOhzL | 0.3 | 80 | 0.4 | 0.1 |
| tOr | 0.6 | 150 | 0.35 | 0.15 |
| zAa | 0.6 | 100 | 0.25 | 0.1 |
| zAf | 0.3 | 40 | 0.25 | 0.25 |
| zAl | 0.5 | 80 | 0.4 | 0.05 |
| zAs | 0.3 | 50 | 0.3 | 0.1 |
| zAw | 0.45 | 80 | 0.4 | 0.05 |
| zLCf | 0.35 | 20 | 0.2 | 0.3 |
| zLCs | 0.3 | 40 | 0.25 | 0.2 |
| zLEw | 0.3 | 80 | 0.3 | 0.05 |
| zLFf | 0.3 | 40 | 0.2 | 0.25 |
| zLFs | 0.3 | 60 | 0.3 | 0.1 |
| zLw | 0.3 | 80 | 0.35 | 0.05 |
| zSAf | 0.3 | 40 | 0.25 | 0.25 |
| zSAl | 0.5 | 80 | 0.35 | 0.05 |
| zSAs | 0.3 | 50 | 0.3 | 0.1 |
| zSAw | 0.3 | 80 | 0.35 | 0.05 |
| zSLCf | 0.35 | 20 | 0.2 | 0.3 |
| zSLCs | 0.3 | 40 | 0.25 | 0.2 |
| zSLFf | 0.3 | 40 | 0.2 | 0.25 |
| zSLFs | 0.3 | 60 | 0.3 | 0.1 |
| zSLw | 0.3 | 80 | 0.35 | 0.05 |
| zVAc | 0.3 | 40 | 0.25 | 0.25 |
| zVAd | 0.3 | 20 | 0.1 | 0.4 |
| zVAl | 0.5 | 100 | 0.35 | 0.15 |
| zVLc | 0.3 | 40 | 0.25 | 0.2 |
| zVLd | 0.3 | 20 | 0.1 | 0.3 |
| zVLl | 0.3 | 80 | 0.3 | 0.05 |
| zXA | 0.5 | 150 | 0.35 | 0.1 |
| zXL | 0.3 | 80 | 0.3 | 0.05 |
| zXx | 0.6 | 200 | 0.15 | 0.3 |
| zYC | 0.3 | 30 | 0.2 | 0.2 |
| zYFf | 0.3 | 30 | 0.2 | 0.25 |
| zYFs | 0.3 | 40 | 0.3 | 0.1 |
| zYFws | 0.3 | 40 | 0.3 | 0.05 |
| zYw | 0.3 | 60 | 0.3 | 0.05 |
| tzAa | 0.6 | 150 | 0.3 | 0.15 |
| tzAl | 0.5 | 100 | 0.35 | 0.1 |
| tzAs | 0.45 | 100 | 0.3 | 0.1 |
| tzAw | 0.3 | 100 | 0.35 | 0.05 |
| tzLEw | 0.3 | 80 | 0.3 | 0.05 |
| tzLFf | 0.3 | 40 | 0.25 | 0.15 |
| tzLFs | 0.3 | 60 | 0.3 | 0.1 |
| tzLw | 0.3 | 80 | 0.3 | 0.05 |
| tzSAl | 0.5 | 100 | 0.3 | 0.1 |
| tzSAs | 0.45 | 100 | 0.3 | 0.1 |
| tzSAw | 0.45 | 100 | 0.3 | 0.05 |
| tzSLw | 0.3 | 80 | 0.3 | 0.05 |
| tzVAl | 0.5 | 150 | 0.35 | 0.1 |
| tzVLd | 0.3 | 30 | 0.15 | 0.3 |
| tzVLl | 0.3 | 80 | 0.3 | 0.05 |
| tzXA | 0.6 | 150 | 0.3 | 0.15 |
| tzYFs | 0.3 | 50 | 0.25 | 0.05 |
| tzYw | 0.3 | 80 | 0.3 | 0.05 |
| bAl | 0.5 | 100 | 0.35 | 0.1 |
| bAw | 0.45 | 80 | 0.35 | 0.05 |
| bLCs | 0.3 | 10 | 0.25 | 0.2 |
| bLEw | 0.3 | 20 | 0.3 | 0.05 |
| bLFf | 0.3 | 5 | 0.2 | 0.25 |
| bLFs | 0.3 | 14 | 0.25 | 0.1 |
| bLw | 0.3 | 40 | 0.3 | 0.05 |
| bSAf | 0.4 | 50 | 0.2 | 0.25 |
| bSLCs | 0.3 | 10 | 0.25 | 0.2 |
| bSLw | 0.3 | 40 | 0.3 | 0.05 |
| bSYFs | 0.3 | 20 | 0.25 | 0.1 |
| bVAc | 0.4 | 40 | 0.25 | 0.2 |
| bVAl | 0.5 | 100 | 0.35 | 0.1 |
| bVLc | 0.3 | 20 | 0.2 | 0.1 |
| bVLl | 0.3 | 80 | 0.3 | 0.05 |
| bXL | 0.3 | 80 | 0.3 | 0.1 |
| bYC | 0.3 | 20 | 0.15 | 0.2 |
| bYFf | 0.25 | 20 | 0.2 | 0.25 |
| bYFs | 0.3 | 40 | 0.25 | 0.2 |
| bYFws | 0.3 | 60 | 0.3 | 0.1 |
| bYw | 0.3 | 60 | 0.3 | 0.05 |
| tbAw | 0.45 | 100 | 0.3 | 0.05 |
| tbLEw | 0.3 | 80 | 0.3 | 0.05 |
| tbLFs | 0.3 | 50 | 0.3 | 0.1 |
| tbLw | 0.3 | 50 | 0.3 | 0.05 |
| tbSAw | 0.45 | 80 | 0.3 | 0.1 |
| tbSLw | 0.3 | 50 | 0.3 | 0.05 |
| tbVAl | 0.45 | 100 | 0.3 | 0.1 |
| tbVLc | 0.3 | 50 | 0.2 | 0.15 |
| tbXA | 0.45 | 150 | 0.4 | 0.2 |
| tbXL | 0.3 | 80 | 0.3 | 0.1 |
| tbYFs | 0.3 | 50 | 0.3 | 0.1 |
| tbYFws | 0.3 | 60 | 0.25 | 0.05 |
| tbYw | 0.3 | 60 | 0.3 | 0.05 |
