# Supplementary material for: S-map parameters for APSIM
Source: MethodsX. 2022 Feb 6;9:101632. doi: 10.1016/j.mex.2022.101632 (PMC8861821; doi:10.1016/j.mex.2022.101632)
Supplement: Supplementary file 2 [file mmc2.docx]

Table S2. Key properties of the two soil profile descriptions used in the APSIM simulations. Volumetric water content at permanent wilting point (θ_PWP_), field capacity (θ_FC_), and saturation (θ_S_), bulk density (ρ), saturated hydraulic conductivity (K_sat_), organic carbon (OC), and fraction of carbon in the organic matter pools BIOM and Inert (FBIOM and FINert).

|  | Soil Depth [cm] | ρ  [Mg/m^3^] | θ_s_  [%] | θ_FC_ [%] | θ_PWP_  [%] | Soil Depth [cm] | OC (%) | FBIOM | FINert |
| --- | --- | --- | --- | --- | --- | --- | --- | --- | --- |
| Horotiu | 0-7.5 | 0.865 | 0.588 | 0.511 | 0.246 | 0-7.5 | 7.259 | 0.08 | 0.561 |
|  | 7.5-15 | 0.835 | 0.605 | 0.451 | 0.207 | 7.5-22 | 5.741 | 0.057 | 0.724 |
|  | 15-30 | 0.805 | 0.616 | 0.439 | 0.203 | 22-32 | 3.942 | 0.019 | 0.849 |
|  | 30-60 | 0.830 | 0.625 | 0.419 | 0.259 | 32-39 | 3.127 | 0.009 | 0.89 |
|  | 60-70 | 0.820 | 0.628 | 0.414 | 0.276 | 39-57 | 2.091 | 0.008 | 0.931 |
|  | 70-100 |  |  |  |  | 57-70 | 1.337 | 0.008 | 0.957 |
|  |  |  |  |  |  | 70-100 | 1.337 | 0.008 | 0.957 |
| Otor_70a.1 | 0-31 | 0.691 | 0.668 | 0.473 | 0.262 | 0-5 | 4.700 | 0.079 | 0.536 |
|  | 31-55 | 0.671 | 0.675 | 0.464 | 0.273 | 5-10 | 4.700 | 0.061 | 0.601 |
|  | 55-73 | 0.735 | 0.651 | 0.446 | 0.241 | 10-15 | 4.700 | 0.048 | 0.656 |
|  | 73-91 | 0.711 | 0.660 | 0.502 | 0.295 | 15-20 | 3.884 | 0.038 | 0.704 |
|  | 91-100 | 0.982 | 0.557 | 0.435 | 0.237 | 20-25 | 2.670 | 0.031 | 0.745 |
|  |  |  |  |  |  | 25-30 | 1.855 | 0.025 | 0.781 |
|  |  |  |  |  |  | 30-35 | 1.310 | 0.021 | 0.811 |
|  |  |  |  |  |  | 35-40 | 0.944 | 0.018 | 0.838 |
|  |  |  |  |  |  | 40-45 | 0.699 | 0.016 | 0.860 |
|  |  |  |  |  |  | 45-50 | 0.534 | 0.015 | 0.880 |
|  |  |  |  |  |  | 50-75 | 0.301 | 0.012 | 0.923 |
|  |  |  |  |  |  | 75-100 | 0.214 | 0.010 | 0.964 |
